# Supplementary material for: Ethanol Production from Wheat Straw Hydrolysate by Issatchenkia Orientalis Isolated from Waste Cooking Oil
Source: J Fungi (Basel). 2021 Feb 6;7(2):121. doi: 10.3390/jof7020121 (PMC7915885; doi:10.3390/jof7020121)
Supplement: Supplementary file 1 [file jof-07-00121-s001.zip › Supplementary Figure S2.pdf]

Supplementary Figure S2

a)

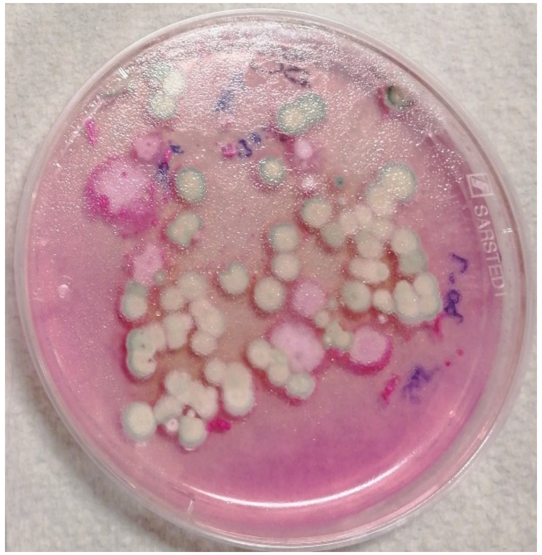

b)

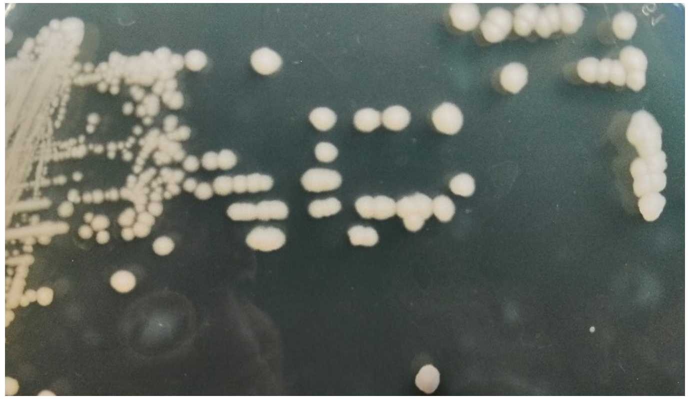

Supplementary Figure S2: Photographic images of isolated microorganisms. a) original agar plate with WCO sample plated on RBC before isolation of single colonies, and b) pure culture of the WCO-isolated *I.orientalis* KJ27-7.
