# Supplementary material for: Ethanol Production from Wheat Straw Hydrolysate by Issatchenkia Orientalis Isolated from Waste Cooking Oil
Source: J Fungi (Basel). 2021 Feb 6;7(2):121. doi: 10.3390/jof7020121 (PMC7915885; doi:10.3390/jof7020121)
Supplement: Supplementary file 1 [file jof-07-00121-s001.zip › Supplementary Figure S3.pdf]

# Supplementary Figure S3

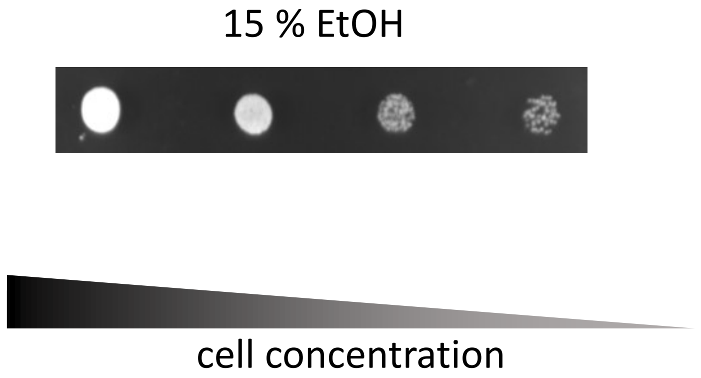


**Supplementary Figure S3:** Spot drop test on 15 % ethanol incubated one day longer. Serial dilutions (1:10, 1:100, 1:1000 and 1:10000) of an *I. orientalis* KJ27-7 cell suspension ($10^8$ cells per ml) were spotted on YNB agar plates containing 20 g $L^{-1}$ glucose and 15 % ethanol (EtOH). Image was taken after 72 h incubation. A representative image of one out of two experiments performed in quadruplicates is shown.
