# Supplementary material for: Ethanol Production from Wheat Straw Hydrolysate by Issatchenkia Orientalis Isolated from Waste Cooking Oil
Source: J Fungi (Basel). 2021 Feb 6;7(2):121. doi: 10.3390/jof7020121 (PMC7915885; doi:10.3390/jof7020121)
Supplement: Supplementary file 1 [file jof-07-00121-s001.zip › Supplementary Figure S4.pdf]

Supplementary Figure S4

a)

b)

c)

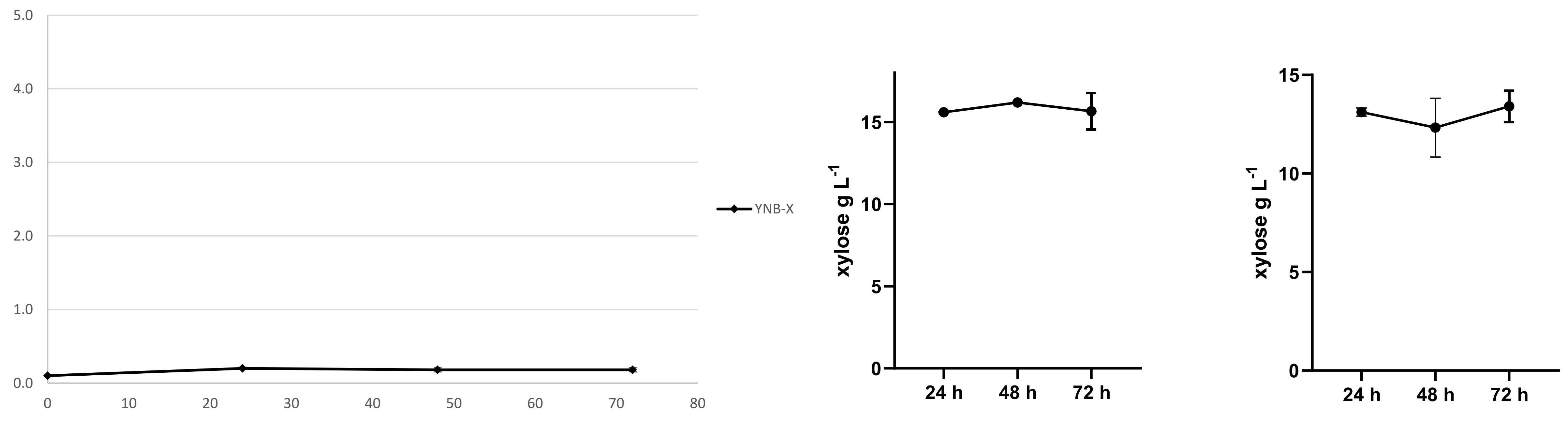

Supplementary Figure S4: Growth on xylose and utilization of xylose by *I. orientalis* KJ27-7. a) OD_{600} of *I. orientalis* KJ27-7 in YNB media with 15 g L^{-1} xylose, measured after 24, 48 and 72 h, and utilization of xylose by *I.orientalis* KJ27-7 as determined by HPLC in YNB media containing b) 15 g L^{-1} xylose and c) 20 g L^{-1} glucose and 13 g L^{-1} xylose as carbon sources.
