# Supplementary material for: Ethanol Production from Wheat Straw Hydrolysate by Issatchenkia Orientalis Isolated from Waste Cooking Oil
Source: J Fungi (Basel). 2021 Feb 6;7(2):121. doi: 10.3390/jof7020121 (PMC7915885; doi:10.3390/jof7020121)
Supplement: Supplementary file 1 [file jof-07-00121-s001.zip › Supplementary Table S1.pdf]

Supplementary Table S1. Composition of WSHL

	**Glucose ($g\ L^{-1}$)**	**Xylose ($g\ L^{-1}$)**	**Acetic Acid ($g\ L^{-1}$)**	**Formic acid ($g\ L^{-1}$)**	**HMF ($g\ L^{-1}$)**	**Furfural ($g\ L^{-1}$)**
**WSHL (bottle 1)**	22.1	9.9	2.0	0.19	0.14	0.05
**WSHL (bottle 2)**	22.1	10.0	1.8	0.15	0.14	0.04
