# Supplementary material for: Ethanol Production from Wheat Straw Hydrolysate by Issatchenkia Orientalis Isolated from Waste Cooking Oil
Source: J Fungi (Basel). 2021 Feb 6;7(2):121. doi: 10.3390/jof7020121 (PMC7915885; doi:10.3390/jof7020121)
Supplement: Supplementary file 1 [file jof-07-00121-s001.zip › Supplementary Figure S1.pdf]

Supplementary Figure S1

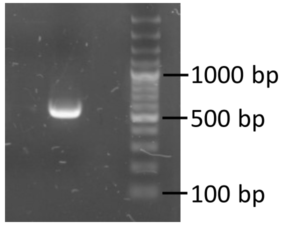

Supplementary Figure S1: PCR-amplified fragment covering ITS1, 5.8S and ITS2 region
The 550 bp fragment was obtained by amplification of the WCO-isolated strains' isolated gDNA with ITS4 and ITS5 primers. The PCR reaction was run on 1 % agarose gel (mV mA 20 min.)
